# From AUC to Action: AI Sepsis Alerts as Quality Improvement Tools in the Emergency Department

**DOI:** 10.5811/westjem.61768

**Published:** 2026-08-21

**Authors:** Abhishek Hanumanpratap Singh Kshatri

**Affiliations:** Apollo Hospitals, Department of Emergency Medicine, Health City, Visakhapatnam, Andhra Pradesh, India

Dear Editor:

Sepsis alert systems in emergency departments (ED) occupy a difficult interface: They are built as prediction models but succeed or fail as clinical decision support and quality improvement (QI) interventions. For that reason, a higher area under the receiver operating characteristic curve (AUC) should not be the endpoint of evaluation. The more relevant question for ED leaders is whether an alert changes care safely: earlier recognition; shorter time to lactate or cultures; faster appropriate antibiotics; acceptable alert burden; and no worsening of overtreatment or inequity.

The ED literature supports both promise and caution. Hwang and colleagues’ *WestJEM* systematic review found that ED sepsis alerts had variable diagnostic accuracy and heterogeneous effects on downstream quality measures, highlighting that implementation design matters as much as detection performance.^1^ More recently, Kim and colleagues’ ED-focused meta-analysis of 22 studies including 19,580 patients found that sepsis alert systems were associated with lower mortality (risk ratio, 0.81; 95% CI, 0.71–0.91) and shorter time to key bundle elements, including fluid administration, blood cultures, antibiotic administration, and lactate measurement.^2^ These findings support alert systems as potentially useful QI tools, but the included studies varied in design, definitions of “time zero,” alert type, and bundled co-interventions.

That heterogeneity is exactly why we should avoid treating artificial intelligence (AI) sepsis alerts as diagnostic oracles. A model can look impressive retrospectively and still fail operationally if it alerts the wrong person, fires too often, lacks a clear expected action, or arrives after clinicians are already concerned. In low-prevalence ED populations, even a model with strong discrimination can generate many false positives, which may create alert fatigue, unnecessary reassessment, broad-spectrum antibiotic overuse, and crowding-related opportunity costs.^2^,^4^ Conversely, an alert with modest AUC may be useful if it fires rarely, identifies patients before clinician suspicion, and reliably triggers sepsis bundle steps.

Implementation of the Targeted Real-time Early Warning System (TREWS), an AI-powered clinical decision support platform, illustrates the point. In a prospective multisite cohort, clinician confirmation of TREWS alerts within three hours was associated with lower adjusted in-hospital mortality (3.3% absolute reduction; 95% CI, 1.7%–5.1%) and earlier treatment among patients whose alerts fired before antibiotics.^3^,^5^ This is encouraging, but it should be described as an adjusted association, not proof that the alert itself caused the outcome difference. Residual confounding remains possible: Clinicians may confirm alerts faster in patients whose clinical trajectory already appears more actionable, and local workflow, clinician engagement, and sepsis governance likely contributed to the observed benefit.

A practical ED evaluation framework should therefore shift from “How accurate is the model?” to “What happened after deployment?” At minimum, AI sepsis-alert pilots should prospectively track the following: (1) time-to-lactate, time-to-cultures, and time-to-antibiotics; (2) proportion of sepsis cases flagged before clinician suspicion; (3) alert volume, false-positive rate, and positive predictive value; (4) clinician response rate and override patterns; (5) antimicrobial stewardship balancing measures; and (6) subgroup performance by age, sex, race/ethnicity, language, and comorbidity when data are available. These metrics convert an AI model from a static classifier into a measurable QI intervention.

Governance should be specified before go-live. Each alert should have a defined recipient, a defined expected response, an escalation pathway, and prespecified thresholds for pausing or retuning the system if alert burden becomes unsafe. Local validation should precede broad deployment because transportability is not guaranteed across institutions, electronic health record builds, patient populations, and sepsis definitions. The pilot should be time-limited, with transparent review of benefit and harm rather than indefinite continuation after technical installation.

There are also important limitations to this framing. Outcome studies of sepsis alert systems are often observational, confounded by co-interventions, and inconsistent in defining sepsis onset and time zero.^1^,^2^ Alert performance depends on base rate and workflow context. Earlier antibiotics are not always better if alerts encourage treatment of noninfectious syndromes or low-risk patients without adequate clinical review. False positives can consume clinician attention and may undermine trust in clinical decision support more broadly.^4^ These limitations do not argue against AI sepsis alerts; they argue for evaluating them as implementation-sensitive QI interventions rather than as self-sufficient diagnostic products.

The reframing is simple: the goal is not a higher AUC; it is safer, faster, measurable sepsis care. The AI sepsis alerts should function as co-pilots for process improvement, not autopilots for diagnosis. Their value should be judged by whether they improve ED workflow and patient outcomes without unacceptable noise, overtreatment, or inequity.
